# Update on Staphylococcal Superantigen-Induced Signaling Pathways and Therapeutic Interventions

**DOI:** 10.3390/toxins5091629

**Published:** 2013-09-24

**Authors:** Teresa Krakauer

**Affiliations:** Department of Immunology, Integrated Toxicology Division, United States Army Medical Research Institute of Infectious Diseases, Fort Detrick, Frederick, MD 21702 5011, USA; E-Mail: teresa.krakauer@us.army.mil; Tel.: +1-301-619-4733; Fax: +1-301-619-2348

**Keywords:** staphylococcal superantigens, SEB, cytokine signaling, PI3K/mTOR, NFκB, FDA-approved drugs

## Abstract

Staphylococcal enterotoxin B (SEB) and related bacterial toxins cause diseases in humans and laboratory animals ranging from food poisoning, acute lung injury to toxic shock. These superantigens bind directly to the major histocompatibility complex class II molecules on antigen-presenting cells and specific Vβ regions of T-cell receptors (TCR), resulting in rapid hyper-activation of the host immune system. In addition to TCR and co-stimulatory signals, proinflammatory mediators activate signaling pathways culminating in cell-stress response, activation of NFκB and mammalian target of rapamycin (mTOR). This article presents a concise review of superantigen-activated signaling pathways and focuses on the therapeutic challenges against bacterial superantigens.

## 1. Overview

*Staphylococcus aureus* produces several exotoxins, staphylococcal enterotoxins A through U (SEA-SEU), and toxic shock syndrome toxin 1 (TSST-1), with potent immunostimulating activities that cause a variety of diseases in humans, including food poisoning, acute lung injury, autoimmune diseases, and toxic shock [1,2,3,4,5,6,7,8,9,10,11,12,13,14,15]. These bacterial toxins were originally known for their enterotoxicity and pyrogenicity. A considerable effort was directed early on at defining their structure and cellular receptors to understand how these toxins exert their biological effects. Staphylococcal exotoxins bind to the major histocompatibility complex (MHC) class II on antigen-presenting cells (APC) and specific regions of Vβ chains of the T-cell receptor (TCR), leading to activation of both APC and T-cells [7,11,14,15,16,17]. The term “superantigen” was coined by Kappler and colleagues in 1989 to describe the novel hyper-stimulatory properties of these bacterial toxins [16]. A decade of crystallographic and structural studies revealed their common molecular structure and binding motifs [18], paving the way for investigations of their signaling mechanisms and the way in which these superantigens exert their potent immunological effects. Unlike conventional antigens, superantigens bypass normal “processing” by APC and induce a large proportion (5%–30%) of T-cells to proliferate at picomolar concentrations [7,16]. The excessive release of proinflammatory cytokines and chemokines from APC, T-cells, and other cell types mediate the toxic effects of staphylococcal superantigens [19,20,21,22,23,24,25]. The proinflammatory cytokines, tumor necrosis factor α (TNFα), interleukin 1 (IL-1) and gamma interferon (IFNγ) have tissue damaging effects [26] and together with matrix metalloproteinases (MMPs) and tissue factor produced by superantigen-activated host cells [27], activate both the inflammatory and coagulation pathways. The increased expression of adhesion molecules and chemokine gradient changes direct leukocyte migration to sites of tissue injury [28]. IL-2 from superantigen-activated T-cells causes vasodilation, vascular leak, and edema [29]. Toxic reactive oxygen species (ROS) from activated neutrophils increase vascular permeability and cause acute lung injury [28]. These molecular changes occur rapidly upon superantigen exposure and progress to hypotension, multi-organ failure and death. In addition to inflammatory pathways activated by staphylococcal superantigens, *S. aureus* also produces numerous virulence factors that aid in its survival and subsequent dissemination in the host. For example, staphylococcal extracellular adherence protein [30] and superantigen-like protein 5 [31] as well as two other staphylococcal surface proteins (the clumping factors A and B) [32] stimulate platelet aggregation which leads to disseminated intravascular coagulation. Targeting the inflammatory and coagulation pathways/molecules represent widely diverse strategies to prevent toxic shock and organ damage resulting from superantigens and various virulence factors [33].

SEB is considered a Category B select agent by the Centers for Disease Control and Prevention (CDC) as it is extremely toxic to humans and can be used as an air-borne, food-borne, and water-borne toxicant. The biodefense objective of mitigation of SEB toxicity in the absence of staphylococcal infection seems simpler when compared to the scenario of replicating pathogens with other virulence factors they produced. Recent efforts have been directed at preventing superantigenic shock, acute lung injury and organ damage resulting from the cumulative biological effects elicited by proinflammatory cytokines. Many reviews and books on superantigens have been published and I will present a concise review on the signaling pathways and give a perspective on the therapeutic modalities for counteracting superantigen-induced shock.

## 2. Staphylococcal Superantigen Structure and Binding to Host Cells

Staphylococcal superantigens are stable, single-chain proteins of 22- to 30-kD that are highly resistant to proteases and denaturation. Despite differences in sequence homology among staphylococcal enterotoxins (SEs) and the streptococcal pyrogenic exotoxins, they have similar protein folds and conserved receptor binding sites [5,15]. These bacterial toxins are classified into five distinct homology groups based on amino acid sequence and similarities in modes of binding to MHC class II molecules [13,15]. Among the different SE “serotypes”, SEA, SED, and SEE share the highest amino acid sequence homology, ranging from 53%–81%, whereas SEB is 50%–66% homologous with SECs. TSST-1 has only a limited sequence homology with other SEs. It has a shorter primary sequence of 194 amino acids with no cysteines, and binds TCR Vβ differently than other SEs [17]. TSST-1 lacks enterotoxicity in non-human primates [34] and has a missing “disulfide loop”, which may be responsible for the emetic activity of SEs, as mutation of residues in this loop abolishes the emetic activity of SEC2 [35]. There is a separation of the emetic and superantigenic domains of SEs since carboxymethylation of histidine residues of SEB resulted in the loss of emetic activity but not superantigenity [36]. Despite varying sequences, structural and crystallographic analysis of SEA, SEB, and TSST-1 show a conserved conformation with two tightly packed domains containing β-sheets and α-helices [18], separated by a shallow groove representing the TCR-binding site [37,38]. The C-terminal domain has a β-grasp motif found in other unrelated proteins. The N-terminal domain contains an oligosaccharide/oligonucleotide-binding (OB) fold, characterized by the presence of hydrophobic residues in the solvent-exposed regions [18].

Superantigens bind to common, conserved elements outside the peptide-binding groove on MHC class II molecules with relatively high affinity (*K*_d_ = 10^−8^–10^−7^ M) [3,39]. Structural analysis shows at least two distinct binding sites on MHC class II molecules for superantigen. A common, low-affinity binding site involving the invariant α-chain of MHC class II and a high-affinity, zinc-dependent binding site on the polymorphic β-chain [39,40,41,42,43,44,45,46]. SEA can cross-link MHC class II molecules on APC by binding to both sites, and persists longer on the cell surface of APC, prolonging its biological effects [47].

The groove formed between the conserved *N*- and *C*-terminal domains of staphylococcal superantigens represents an important interaction site for the TCR Vβ chain [48,49,50,51]. Each superantigen binds to a distinct repertoire of Vβ-bearing T-cells, revealing a unique biological “fingerprint” which might be useful for diagnosing toxin exposure [51,52]. The binding of superantigens to the Vβ chain of TCR is of low affinity (*K*_d_ = 10^−4^–10^−6^ M), with contacts mostly between the side-chain atoms of the superantigen and the complementarity-determining regions 1 and 2 and the hypervariable region 4 of the Vβ chain. Studies with mutants of SEB and SEC3 indicate that a small increase in the affinity of a superantigen for MHC can overcome a large decrease in their affinity for the TCR [48]. Thus, the multiple modes of superantigen binding to MHC and TCR indicate a cooperative effect of interactions in the formation of the trimolecular complex, hyper-activating the host immune system. The superantigen/MHC interactions strengthen their binding to TCR such that they mimic TCR binding to a conventional MHC-peptide complex [49]. Other co-stimulatory receptors on both cells also interact to further stabilize superantigen binding to many cell types [53,54]. A direct binding of SEB to the T-cell co-stimulatory receptor CD28 was reported recently [55]. Peptides derived from the CD28 binding region protected mice from SEB-induced lethality and reduced TNFα, IL-2 and IFNγ expression [55]. This correlates with previous reports of the resistance of CD28-deficient mice to superantigen-induced shock and the lack of serum TNFα and IFNγ after toxin challenge in these mice [56,57].

## 3. Three Signals Synergize to Sustain Cell Activation

The three signals required for T-cell activation by superantigens and conventional antigens are similar even though superantigens bind outside the peptide-binding groove of MHC class II molecules. The first signal is induced upon the binding of superantigen with TCR-CD3 complex, which activates the Src family of protein tyrosine kinases (PTKs) [58,59,60]. The engagement of co-stimulatory molecules on APC and T-cells, subsequent to superantigen binding, results in a second signal that optimizes and sustains T-cell activation [61,62,63]. The interactions between the adhesion molecules LFA-1 with intercellular adhesion molecule 1 (ICAM-1), and the co-stimulatory molecules CD28 with CD80 on T-cells and APC, respectively, promotes stable cell conjugates. Co-ligation of receptors results in extensive cytoskeletal remodeling and the formation of immunological synapse, initiating signaling cascades [61,64]. PTKs, including Lck and ZAP-70, phosphorylate tyrosine-based motifs of the TCR intracellular components and other adaptors [58,59,65]. The TCR-induced kinases activate phospholipase C gamma (PLCγ) resulting in the generation of second messengers and increase in intracellular calcium levels. One specific second messenger, diacylglycerol (DAG), subsequently activates protein kinase C (PKC) and the proto-oncogene Ras [64,66]. PKC activates downstream signaling pathways including the mitogen-activated protein kinase (MAPK) and the NFκB cascade [67]. Many proinflammatory cytokine genes contain NFκB binding sites in the promoter region and are activated by NFκB [68]. The cytokines IL-1, TNFα, IFNγ, IL-2 and IL-6, and chemokines, in particular, MCP-1, are induced directly by superantigens *in vitro* and *in vivo*. The inflammatory environment provided by these proinflammatory mediators represents the third signal for T-cell activation. IL-1 and TNFα activate many other cell types including fibroblasts, epithelial, and endothelial cells to produce other mediators, cell adhesion molecules, tissue protease MMPs, and ROS. IFNγ from superantigen-activated T-cells activates expression of MHC class II and adhesion molecules, and synergizes with IL-1 and TNFα to promote tissue injury, specifically in the gut [10]. Collectively and individually, these mediators from superantigen-activated cells exert damaging effects on the immune and cardiovascular systems, culminating in multi-organ failure and lethal shock.

## 4. Cross-Talk among Key Signaling Pathways

**Figure 1:**
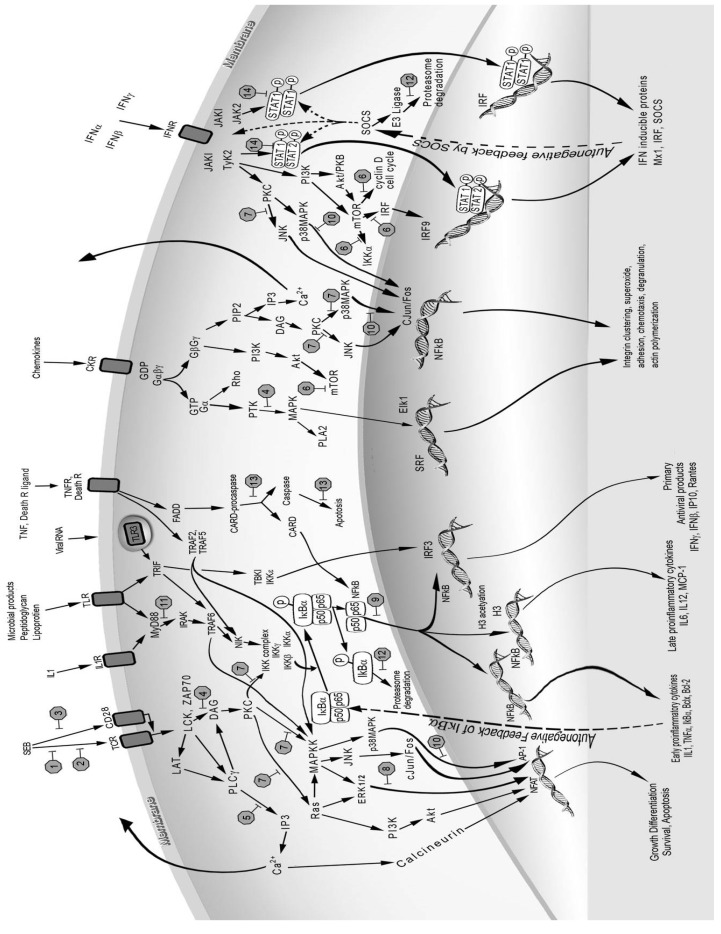
Cell receptors, intracellular signaling molecules, and signal transduction pathways used by superantigens and mediators induced by superantigens. Potential targets of inhibition are represented by stop signs 1–14, numbered in order of their description in the text. 1. Major histocompatibility complex (MHC) class II (not shown); 2. T-cell receptor (TCR) Vβ; 3. CD28; 4. Tyrosine kinases; 5. Phospholipase C (PLC); 6. Mammalian target of rapamycin (mTOR); 7. Protein kinase C (PKC); 8. Extracellular signal-regulated kinase (ERK1/2); 9. NFκB; 10. p38 MAPK; 11. Myeloid differentiation factor 88 (MyD88); 12. Proteasomes; 13. Caspases; 14. Signal transducer and activator of transcription (STAT).

The three signals of T-cell activation exert their potent effects by activating the phosphoinositide 3 kinase (PI3K)/mammalian target of rapamycin (mTOR), NFκB and MAPK pathways [67,68,69]. A description of these signal transduction pathways upon superantigen binding to host cell receptors was presented recently (Figure 1) [70]. Phosphorylation and dephosphorylation events modulate all three cascades with specific kinases and phosphatases. PTKs and lipid molecules from PLCγ activation trigger the PI3K pathway upon specific ligand binding to a number of receptors besides the TCR. Co-stimulatory receptor CD28, IL-2 receptor (IL-2R), IFNγR, growth factor receptor, and G-protein-coupled receptor (GPCR) all activate PI3K [69]. Different PTK inhibitors including genistein, tyrphostin, and herbimycin A, reduced IL-1 levels in TSST-1-stimulated cells [65]. PI3K activates Akt (also known as PKB) and mTOR downstream and modulates many biological processes including cell growth, differentiation, proliferation, survival, migration and metabolism [71,72,73,74,75,76]. The importance of the PI3K/mTOR pathway is shown by the efficacy of rapamycin, a specific inhibitor of mTOR complex 1 (mTORC1), in protecting mice from SEB-induced lethal shock [77]. Rapamycin inhibited SEB-stimulated T-cell proliferation and reduced SEB-induced IL-2 and IFNγ *in vitro* and *in vivo*. An alternative pathway of T-cell activation by SEE bypasses PTK tyrosine phosphorylation and uses PLCβ to activate PKC, ultimately activating extracellular signal-regulated kinase 1 and 2 (ERK1/2), NF-AT and NFκB [78].

The MAPK pathway is induced by mitogens, superantigens, cytokines, chemokines, growth factors, as well as environmental stress, and comprises of three major kinase cascades, ERK1/2, c-Jun-*N*-terminal kinase (JNK), and p38 MAPK. These MAP kinases control fundamental cellular processes to signal cell stress, culminating in the activation of transcription factors NFκB, NF-AT and AP-1 [79], affecting proliferation, differentiation and apoptosis. One common upstream activator of the MAPK pathway is PKC which is activated by TCR, co-stimulatory receptors and GPCR. MAPK promotes inflammation by targeting NFκB to promoters of inflammatory genes [80]. IL-1 and TNFα are both activators and effectors of MAPKs, as these mediators both activate MAPK via various intracellular TNF receptor-associated factors (TRAFs), and are themselves induced by MAPK activation.

The proinflammatory cytokines IL-1 and TNFα can directly activate the transcriptional factor NFκB in many cell types that include epithelial and endothelial cells. IL-1 interacts with IL-1 receptor 1 (IL-1R1) and receptor accessory protein, uses signaling molecules, the adaptor myeloid differentiation factor 88 (MyD88), IL-1R-associated protein kinase 1 (IRAK1), and TRAF-6 to activate IκB kinases (IKK), leading to NFκB activation [81]. Phosphorylation of the inhibitory protein IκBα by IKK leads to IκBα degradation and release from cytoplasmic NFκB. This allows NFκB to translocate to the nucleus where it binds to promoter regions of various inflammatory genes [82]. Activation of NFκB leads to induction of many proinflammatory and anti-apoptotic genes. IL-1R1 has structural homology to toll-like receptors (TLRs) which use similar intracellular adaptors and molecules as those used for IL-1R1 for signaling (Figure 1). TLRs are receptors used by the host to sense pathogen associated molecules such as lipoprotein, peptidoglycan, lipopolysaccharide, flagellin, dsRNA and viral RNA to activate a rapid innate response [83]. Recently, SEB was shown to upregulate the expression of TLR2 and TLR4, thereby enhancing the host response to other microbial products [84,85,86]. This might partially account for the synergistic effects of LPS and SEB in mouse models of SEB-induced shock [87,88,89].

TNFα binds to TNF receptor 1 and 2 (TNFR1, TNFR2) and signals with different intracellular TRAFs, ultimately activating MAPK and NFκB, and results in the expression of other cytokines, adhesion and co-stimulatory molecules [26,90]. An important and damaging component of signaling by the TNFR superfamily which includes various death receptors is caspase activation via the intracellular death domains of these TNFRs. Receptors in this superfamily use intracellular adaptors, TNFR-associated death domain (TRADD) and Fas-associated death domain (FADD) to activate the caspase 8 cascade, JNK, and NFκB. These multiple pathways account for the pleiotropic effects of TNFα including apoptosis, cell activation, coagulation, inflammation, and host defense [90]. The synergistic effects of TNFα and IFNγ on epithelial cells increase ion transport, leading to cell damage and epithelial leakage [10]. The critical role of TNFα in mediating lethality was shown by anti-TNFα antibodies protecting mice from SEB-induced shock in a D-galactosamine (Dgal)-sensitized model [91].

Chemokines, and T-cell cytokines, IFNγ and IL-2, bind to their respective receptors and activate the PI3K/mTOR and MAPK pathways with diverse signal transducers. IFNγ binds to IFNγR, uses Janus kinase 1 and 2 (JAK1, JAK2) to phosphorylate the signal transducer and activator of transcription 1 (STAT1) [92,93]. The main function of IFNγ is in antimicrobial defense as it activates antiviral genes, adhesion molecules, immunoproteasome, and E3 ligase. The IFNγ-activated JAKs also activate PI3K/mTOR independent of STAT1 [94]. Additionally, IFNγ induces the expression and activation of death receptors including Fas (CD95), leading to cell apoptosis [95]. Thus, IFNγ-induced immunoproteasome and CD95 death signaling pathways contribute to vascular cell apoptosis and cardiovascular inflammation [95]. The death receptors use intracellular death domains to activate FADD and caspase 8, resulting in mitochondrial cytochrome c release and DNA fragmentation. IFNγ disrupts ion transport and barrier function in superantigen-activated epithelial cells and these biological effects are amplified by TNFα [96]. However, anti-IFNγ had no effect on mortality and only reduced SEB-induced weight loss and hypoglycemia in the Dgal-sensitized mouse model of lethal shock [97]. A recent study suggests that IFNγ from SEB-stimulated cells plays an important role in autoimmunity in HLA-DQ8 transgenic mice [98].

IL-2 binds to the IL-2R and signals through JAK1 and JAK3 to activate PI3K and Ras, affecting proliferation, growth, and differentiation of many cell types [99]. Ras signals through the MAPK pathway to activate AP-1, cJun/Fos and NFAT. IL-2 increases microvascular permeability and induces vasodilation, resulting in perivascular edema in SEB-induced lung injury [100,101]. IL-2-deficient mice are resistant to SEB-induced toxic shock [102].

IL-6, from both macrophages and activated T-cells, has some overlapping activities with IL-1 and TNFα, and activates JAK3 and Ras upon binding to IL6R [103]. Additionally, IL-6R also signals through PI3K/mTOR to promote cell survival. The Ras pathway used by IL6, IL2, IFNγ, TCR and co-stimulatory receptors results in MAPK activation whereas the alternate PI3K pathway activates mTOR.

The chemokines IL-8, MCP-1, MIP-1α, and MIP-1β, are induced directly by SEB or TSST-1 and are potent chemoattractants and leukocytes activators [22,26,104]. Chemokines bind to seven-transmembrane GPCR, induce early calcium flux, activate PLC and signal via the PI3K/mTOR pathway [26,104,105]. Chemokines orchestrate leukocyte migration to promote inflammation and increase tissue injury. Exudates from superantigen-injected air pouches contained predominantly neutrophils with few macrophages [22]. Recruited- and activated-neutrophils produce cytotoxic superoxide and MMPs, contributing to organ damage. Systemic or intranasal exposure to SEB resulted in acute lung injury characterized by increased expression of adhesion molecules ICAM-1 and VCAM, increased neutrophil and mononuclear cell infiltrate, endothelial cell injury, and increased vascular permeability [28,106].

TCR, co-stimulatory receptors and cytokines signal with diverse intracellular molecules to activate PI3K/mTOR, MAPK, and IKK/NFκB cascades. There is cross-talk among these pathways as the MAPKs cascade downstream from TCR, co-stimulatory receptors and T-cell cytokines all activate NFκB, whereas TRAFs from IL-1 and TNFα signaling activate MAPK and NFκB independently. There is some overlap and redundancy of these activation pathways as multiple receptors activate PI3K/mTOR, MAPK and NFκB. However, specificity exists as illustrated by the different classes of MAPKs and their targets. JNK regulates c-Jun and AP-1, and has detrimental effects in the liver whereas p38 MAPK has an additional effect on the phosphorylation of eukaryotic initiation factor (eIF-4E) and promotes translation [79]. The cellular responses to individual cytokines are also different and specific with IFNγ increasing cellular permeability in activated epithelial and endothelial cells whereas IL-1 has prothrombotic effects on the endothelium through the increased production of tissue factor and prostaglandins.

## 5. Mouse Models of Superantigen-Induced Shock

Superantigens from *S. aureus* and *Streptococcus pyogenes* are the causative agents of serious life threatening toxic shock syndrome (TSS) and the excessive release of cytokines contributes to the pathogenesis of TSS [1,2,3,33]. SEB has historically been used as a prototype superantigen in biological and biodefense research investigations, as humans are extremely sensitive to SEB especially by inhalation. An obvious step in developing new therapeutic approaches for SEB-induced toxic shock is finding relevant models that mimic human disease.

Mice are often used as a model to study the immunological mechanisms of superantigen mediated shock [21,22,25,28,55,56,57,87,88,89,101]. Although these animals lack an emetic response, they are ideal to work with as immunological reagents are available, the strains and genetic backgrounds including specific MHC class II are well-defined, and the low cost of maintenance allows more animals to be used in experimental protocols. However, mice are naturally less susceptible to SEs when compared to humans because of the lower toxin affinity to murine MHC class II [88,107]. As a result, mice do not develop lethal SEB shock and potentiating agents such as Dgal, actinomycin D, LPS, or viruses are used together with toxin to induce toxic shock [88,91,108,109,110,111]. Depending on the injury model, sensitizing agents and route of delivery, the severity of disease may involve different organs and distinct profile of mediators. Both Dgal and actinomycin D induced TNFα-dependent hepatotoxicity, and SEB-induced shock models using these potentiators showed much higher serum levels of TNFα not present when SEB was used alone and liver injury was a key feature in these models. Both IL-2 and IFNγ are also critical in Dgal-sensitized models of superantigen-induced shock as IL-2 deficient mice were resistant to SEB-induced shock and antibodies to IFNγ inhibited SEB-induced weight loss and hypoglycemia [97,102].

LPS, a cell wall component of gram negative bacteria, is the most frequently used synergistic agent in mouse model of SEB-induced shock [25,87,88,89,111]. Relatively high doses of SEB or LPS are used together in these models, usually with Balb/c mice. The shock syndrome induced by superantigens in these models results from the culmination of the biological effects of elevated levels of IL-1, TNFα, and IFNγ, not seen in the absence of LPS [88]. An analysis of the interdependent effects of various doses of SEB used alone and together with LPS in different dose combinations indicated that prolonged levels of certain cytokines were necessary to induce lethal shock in Balb/c mice [25]. Non-survivors in SEB plus LPS groups have significantly higher levels of TNFα, IL-6, MIP-2, and MCP-1 early (eight hours) after SEB exposure [25]. In addition to these mediators, non-survivors showed higher levels of IFNγ and IL-2 later at 24 h. In this LPS-sensitized shock model, lethality and cytokine response were both influenced mostly by the LPS dose and not by SEB. The early TNFα release and sustained levels of IFNγ, IL-2, IL-6, MIP-2 and MCP-1 later correlated with acute lethal shock at 48 h. Since LPS and SEB activate similar cytokines and cells, although using different receptors, it is difficult to compare the molecular mechanisms of shock in this traditional model with human TSS.

To avoid the confounding effects of potentiating agents, a “double-hit” low dose SEB model was developed in a LPS resistant mouse strain C3H/HeJ to simulate human TSS [101]. This model mimics human TSS as intranasal delivery of SEB triggers lung inflammation, systemic release of cytokines, and hypothermia that culminate in death at later time points unlike the various potentiated models with much earlier lethal end points. An alternative model using transgenic mice expressing human HLA class II molecules was established to recapitulate human TSS, with different susceptibility to various superantigens dependent on the inserted human HLA class II type, DQ or DR [112,113,114,115,116]. Transgenic mice expressing HLA-DQ6 still required Dgal to potentiate the effects of SEB [112] whereas mice with HLA-DR3 were sensitive to SEB alone [116]. A recent study revealed multiple organ inflammation in lung, liver, kidney, heart and the small intestine that accompanied lethal shock in HLA-DR3 transgenics [116]. Moreover, intestinal absorptive functions were also interrupted in this transgenic model of SEB-induced shock.

## 6. Vaccines and Therapeutic Antibodies

There is currently no available treatment for superantigen-induced shock except for the use of intravenous human immunoglobulin [117]. Antibodies to superantigens can provide broad spectrum protection as neutralizing antibodies against one superantigen cross-react and block the biological effects of a different superantigen [118]. Naturally protective antibodies against superantigens can be found in *S. aureus* bacteremia and increase in neutralizing titers during infection correlated with recovery [119]. Other studies showed that there is a correlation of lower serum antibodies to TSST-1 in patients with recurring TSS [120,121]. Both monoclonal and human-mouse chimeric antibodies against SEB with high affinities in the picomolar range have been used effectively to target SEB-induced host responses [122,123,124]. The use of antibodies has certain limitations since neutralization of toxins is effective only at the early stages of exposure as it blocks the first step of host receptor interaction before cell activation. Vaccination is a proven method to prevent SEB-induced shock and attenuated mutants of SEB with defective MHC class II binding which lack superantigenicity were efficacious against toxin challenge in mice, piglets and monkeys [125,126].

## 7. Inhibitors of Cell Receptor-Toxin Interaction

A number of small overlapping peptides, encompassing a conserved region of SEB (residues 150–161), bind to host cell receptors and have been tested to block superantigen-induced effects both *in vitro* and *in vivo* with contradictory results using the same peptide [111,113,127]. Although the dodecapeptide prevented transcytosis of various SEs across human intestinal epithelial cell monolayer and may block co-stimulatory signals [128], this and other “SEB peptide antagonists” failed to block SEB-induced T-cell proliferation in human peripheral blood mononuclear cells (PBMC) and had no effect on SEB-induced lethal shock in HLA-DR3 transgenic mice [113]. Blockade of the CD28 co-stimulatory receptor by its synthetic ligand, CTLA4Ig (also known as abatacept) prevented TSST-1-induced shock, and reduced the serums TNFα, IL-2, and IFNγ [129]. Peptide mimetics of CD28 also prevented co-stimulatory receptor interaction with SEB and inhibited SEB effects *in vitro* and *in vivo* [55]. Furthermore, a specific CD28-peptide mimetic blocked superantigen-binding to CD28 and attenuated toxic shock and necrotizing soft-tissue infection induced by *Streptococcus pyogenes* [130]. Anti-CD44 reduced the binding of SEB-activated leukocytes to lung epithelial cells and prevented acute lung injury [131]. Bi-specific chimeric inhibitors composed of MHC class II and TCR Vβ domains competitively blocked SEB binding and cell activation in human PBMC [132]. A soluble TCR Vβ mutant also neutralized the effects of superantigens *in vitro* [133]. Blocking receptor interaction has many limitations as blockade of host receptors might adversely affect immune function and the inhibitor has to be administered pre-exposure or soon after exposure to toxins for it to be effective. Aside from cross-reactive antibodies, receptor blockade inhibitors are usually specific and have to be tailor-made for a specific superantigen [132]. A list of the therapeutics used in mouse SEB-induced toxic shock models is shown in Table 1, arranged in order of their description in the text.

## 8. Inhibitors of Signal Transduction and Cytokines

Targeting host responses after superantigen exposure is an attractive strategy as these events occur later and may be more amenable to interruption. An important class of therapeutic compounds to be considered is inhibitors that can block signal transduction pathways activated by superantigens. Inhibitors of signal transduction are often cytokine inhibitors as cytokines are the best known “signalers”, acting both as activators and effectors. An obvious advantage of signal transduction inhibitors is that they can be administered post-exposure and are likely broad spectrum, inhibiting many different superantigens or even pathogens that trigger similar host responses and signaling pathways. The pathways central to superantigen activation are PI3K/mTOR, MAPK, and NFκB, which are also used by other pathogens, TLRs, and cytokines.

Various animal models indicated TSS results from the cytokine signals activating host pathways and inducing damage in multiple organs. The convergence of multiple receptor signaling allows activation of innate host pathway signals to persist and dominate, and these “signals” are likely potential therapeutic targets. An example is NFκB, which binds to the promoter regions of many inflammatory genes implicated in TSS including cell adhesion molecules, cytokines, chemokines, acute phase proteins, and inducible nitric oxide synthase [82]. The downstream activation of NFκB leads to the inducible expression of mediators involved in inflammation and tissue injury seen in SEB-induced lung injury and toxic shock models. A cell-permeable cyclic peptide targeting NFκB nuclear transport prevented lethal shock in Dgal-sensitized mice accompanied by attenuation in liver apoptosis and hemorrhagic necrosis [100,134]. This NFκB inhibitor also reduced SEB-induced inflammatory cytokines and T-cell responses [100].

**Table 1:** Therapeutics tested for efficacy in murine models of staphylococcal enterotoxins (SEB)-induced toxic shock. * indicates drug is FDA-approved.

Pharmacologic agent	Target	Biological effects against SEB
Anti-SEB monoclonal antibodies	SEB	Neutralized mitogenic activity of SEB *in vitro*. Prevented SEB-induced shock in HLA-DR3 transgenic mice [124].
SEB-peptide antagonists	MHC	Blocked superantigen binding to MHC class II in human PBMC and inhibited T-cell proliferation [111]. Afforded 83% protection in mouse model of SEB + LPS-induced shock [111]. Failed to block SEB-induced T-cell proliferation in human PBMC [113]. No protective effect against SEB-induced shock in HLA-DR3 transgenic mice [113]. Decreased SEB-induced IL-2, IFNγ and TNFβ gene expression [127]. Protected 80% of Dgal-sensitized mice against SEB-induced shock [127].
Mimetic peptides of CD28	CD28	Blocked superantigen binding to CD28 and attenuated SEB-induced IL-2, TNFα, and IFNγ [55]. Protected mice from SEB-induced shock [55,130].
Cell-permeable peptide targeting NFκB	NFκB nuclear translocation	Attenuated serum TNFα, IFNγ and IL-6. Protected mice from liver injury and SEB-induced shock in Dgal-sensitized mice [134].
Dexamethasone *	NFκB	Inhibited SEB-induced proinflammatory cytokines and chemokines in human PBMC. Reduced serum levels of cytokines, attenuated hypothermia due to SEB, and protected mice 100% in both SEB-induced and SEB + LPS-induced shock models [106,135].
Bortezomib *	NFκB, proteasome	Decreased serum cytokine but no effect on lethality in HLA-DR3 transgenic mice challenged with SEB [136].
Mimetic peptides of BB loop of MyD88	MyD88	Reduced SEB-induced IL-1β, TNFα and IFNγ. Provided 83% protection in SEB + LPS-induced shock model [137,138].
D609	PLC	Blocked SEB-induced cytokines and chemokines [139]. Protected 90% of mice from SEB-induced lethal shock [140].
Cell-permeable SOCS3	STAT1	Inhibited cytokine production, attenuated liver necrosis, and prevented SEB + LPS-induced lethal shock [141].
Rapamycin *	mTORC1	Blocked SEB-induced cytokines and chemokines. Protected mice 100% from lethality even when administered 24 h after SEB [77,142].
Tacrolimus *	Calcineurin phosphatase	Suppressed serum cytokines but no protection against SEB-induced shock in HLA-DR3 transgenic mice [143].
*N*-acetyl cysteine *	NFκB, ROS	Suppressed NFκB activation but protected only 30% of mice from SEB-induced lethal shock [144,145].
Dexamethasone * + *N*-acetyl cysteine *	NFκB, ROS	When used in tandem, reduced SEB-induced cytokines, hypothermia, and protected 75% of mice from lethal shock [145].
Niacinamide	Nitric oxide	Reduced SEB-induced cytokines and lethality in SEB + LPS-induced shock model [146].
Pentoxifylline *	Phospho-diesterase	Attenuated SEB-induced cytokines *in vitro* and *in vivo*. Prevented lethality in SEB + LPS-induced shock model [147].

There are other NFκB inhibitors which are FDA-approved for treatment of inflammatory diseases and cancers [148,149]. Dexamethasone, an immunosuppressive corticosteroid, potently attenuated superantigen-induced T-cell proliferation, cytokine release, and cell activation marker expression in human PBMC [150]. Dexamethasone also prevented lethal shock accompanied by attenuation of the hypothermic response, weight loss and serum cytokines in the LPS-potentiated SEB model and the SEB “double-hit” model of toxic shock [106,135]. The pulmonary lesions were reduced by dexamethasone treatment only at later time points (96 to 168 h) and resolution of lung inflammation lagged behind the reduction in cytokines such that a long course of steroid treatment was necessary to rescue mice from lethal shock [106]. Bortezomib, another inhibitor of NFκB, and a proteasome inhibitor, blocked SEB-induced cytokine release but had no effect on lethality or liver necrosis in transgenic mice [136]. Natural products such as epigallocatechin gallate (EGCG) from green tea, and resveratrol (RES) from red wine, are also NFκB inhibitors that separately reduced superantigen-induced T-cell proliferation and cytokine release from human PBMC [151]. EGCG attenuated IFNγ-induced epithelial permeability increases and suppressed T-cell activation and cytokines from SEB-stimulated human PBMC and murine lymph node cells [152]. RES reduced lung injury by blocking SEB-induced T-cell activation, pulmonary permeability increases, and caspase 8-dependent apoptosis [153]. Another upstream inhibitor of NFκB, a synthetic mimetic (EM-163) to the BB-loop of MyD88, reduced multiple cytokines in superantigen-stimulated human PBMC and protected mice from lethal shock in the LPS-sensitized model [137,138]. However, the complete or long-term blockade of NFκB would likely produce adverse side effects as NFκB is essential in maintaining normal host defense and homeostasis [68,154].

Other pathway inhibitors include those directed against the various kinases, PKC, MAPK and PTK. Genistein, a tyrosine kinase inhibitor, and H7, a PKC inhibitor, separately reduced TNFα but not IL-1 from TSST-1-stimulated PBMC [155]. A selective inhibitor of p38 MAPK (SB203580) and an inhibitor of ERK (PD098059) each partially blocked TNFα production from SEB-stimulated human T cell clones [156]. D609, an inhibitor of PLC, which is downstream from superantigen binding to TCR and CD28, blocked SEB-induced effects both *in vitro* and *in vivo* [139,140]. SOCS3, an intracellular feedback inhibitor of the various STATs used by IFNγ and IL-2 signaling, reduced the effects of these two cytokines [92]. A cell-permeable form of SOCS3 reduced the lethal effects of SEB and LPS by inhibiting the production of inflammatory cytokines and attenuating liver apoptosis and hemorrhagic necrosis [141].

Immunosuppressive drugs are also good candidates to block superantigen-induced immune responses as they are potent inhibitors against many cell types including T-cells and macrophages. Three FDA-approved drugs for preventing transplant rejection have been used in three different animal models of SEB-induced toxic shock. Cyclosporine A (CsA) inhibited SEB-induced T-cell proliferation *in vitro*, reduced serum cytokines, and attenuated pulmonary inflammation, but has no effect on lethality in monkeys [157]. Rapamycin, a specific inhibitor of mTORC1, was efficacious even when given 24 h after SEB in the SEB “double-hit” model [77]. Rapamycin blocked SEB-induced T-cell proliferation, reduced serum cytokines, and prevented hypothermia and weight loss induced by SEB. Intranasal rapamycin also protected mice against SEB-induced shock when administered as late as 17 h after toxin exposure, providing a practical route of drug delivery against SEB [142]. Another structural analog of rapamycin, tacrolimus, suppressed superantigen-induced T-cell activation *in vitro* but did not reduce lethality in HLA-DR3 transgenic mice [143].

Another hallmark of SEB-intoxication is acute lung injury which is most likely a result of oxidative stress inducing damage in the lung. Acute lung injury arises as SEB-, cytokine- and chemokine-activated neutrophils migrate into lung areas producing high levels of superoxide, which is capable of inducing vascular permeability and apoptosis [28]. The anti-oxidants *N*-acetyl cysteine (NAC) and pyrrolidine dithiocarbamate (PDTC) each mitigated NFκB signaling and T-cell proliferation, and blocked cytokine production in superantigen-activated human PBMC [144]. However, NAC has only a minor effect *in vivo*, reducing lethality by 30% in the SEB “double-hit” model [145]. Dexamethasone, although effective against SEB-induced shock, required a prolonged dosing of up to four days, which might not be ideal in a clinical setting as dexamethasone is immunosuppressive. Treatment with a short course of dexamethasone (up to five hours post-SEB) provided only 20% protection. Importantly, the combined effects of a short treatment course of intranasal dexamethasone followed by NAC prevented SEB-induced shock, hypothermia and weight loss [145]. Both dexamethasone and NAC are FDA-approved drugs that act distal to toxin binding. Another combination treatment, using a human-mouse chimeric anti-SEB antibody and lovastatin concomitantly and immediately after toxin exposure, also protected transgenic mice from SEB-induced shock [158].

Most therapeutic testing in animal models of SEB-induced shock have targeted proinflammatory cytokines as there is a strong correlation between toxicity and elevated serum levels of these mediators [21,22,23,24,25]. Inhibitors aimed at blocking proinflammatory mediator release overlap with inhibitors of signal transduction triggered by superantigens. The critical role of TNFα in lethal shock was established by the prevention of SEB-induced lethality with neutralizing antibodies against TNFα in Dgal-sensitized mice [91]. IL-10, an anti-inflammatory cytokine, prevented superantigen-induced toxic shock by reducing the production of the proinflammatory mediators IL-1, TNFα and IFNγ [159,160]. The nitric oxide inhibitor niacinamide improved the survival of mice given LPS plus SEB by attenuating serum IL-2 and IFNγ [146]. Doxycycline, an antibiotic, inhibited SEB-induced T-cell proliferation, proinflammatory cytokines, and chemokines in human PBMC [161]. Recently, a panel of different antibiotics was tested for inhibitory effects on cytokine release from SEA- and TSST-1-stimulated human PBMC [162]. Tigecycline decreased IL-6 and IFNγ whereas trimethoprim increased IL-8 and TNFα from superantigen-stimulated cells. Clindamycin, daptomycin, vanomycin and azithromycin had no effect on cytokine release in these stimulated cells. Another study showed that azithromycin suppressed TSST-1-induced T-cell proliferation by blocking ERK and JNK activity [163]. Pentoxyfylline, a phophodiesterase inhibitor used clinically to treat peripheral vascular disease, reduced cytokines and T-cell proliferation in SEB- or TSST-1-stimulated cells [147]. Pentoxyfylline prevented lethal shock accompanied by a reduction in serum cytokines in the LPS plus SEB mouse model [147].

Caspase inhibitors have also been used to attenuate the toxic effects of superantigens as caspases initiate cellular apoptosis and the release of certain cytokines from inactive precursors. The release of IL-1β is dependent on caspase 1, a proteolytic enzyme that cleaves pro-IL-1 into active IL-1β [26]. The caspase 1 specific inhibitor, Ac-YVAD-cmk, attenuated both IL-1β and MCP-1 production in SEB- and TSST-1-stimulated PBMC cultures but had no effect on other cytokines or T-cell proliferation [164]. Caspase 3 and caspase 8 are enzymes involved in SEB-induced cell apoptosis but inhibitors of these two caspases were ineffective in reducing superantigen-induced cytokines or T-cell proliferation [164]. In contrast, a pan-caspase inhibitor, Z-D-CH_2_-DCB, blocked the production of IL-1β, TNFα, IL-6, IFNγ, MCP-1, MIP-1α, MIP-1β, and inhibited T-cell proliferation in SEB- and TSST-1-stimulated PBMC [164].

## 9. Repurposing of FDA-Approved Drugs for Biodefense Agents

As seen from the above studies, FDA-approved drugs currently used for other indications including dexamethasone, rapamycin, cyclosporine A, tacrolimus, bortezomib, doxycycline, pentoxyfylline, NAC, PDTC, have been tested as therapeutics against superantigens with varying degree of success since the 1990s. The testing of FDA-approved drugs for preventing superantigen-induced shock should speed up the approval process for biodefense use in case of exposure. However, as seen from the various FDA-approved drugs tested, even knowing the mechanism of action of these drugs is no guarantee for success as *in vivo* dosages, dosing routes and schedules as well as animal models all affect the outcome. Rapamycin, by decreasing the levels and effects of IL-2 and IFNγ through mTOR inhibition, is proven to be the most effective single agent to counter both intranasal and systemic exposure to SEB [77,142].

Repurposing of FDA-approved therapeutics makes sense for biodefense use as the therapeutics approval process for human use requires resources and time that might not work for biodefense-related agents. Currently, the approval rate for therapeutics through the FDA is low with 90% of drugs rejected due to safety concerns, inadequate bioavailability or lack of efficacy [165,166]. Intuitively, drug repurposing makes use of the drug’s mechanism of action and applies it to diseases or bioterror agents with known or putative pathogenic effects. It bypasses the usual time and resource consuming process of target discovery, optimization, preclinical development and clinical safety testing, and might possibly obtain faster regulatory review by the FDA. This fast track method of repurposing FDA-approved drugs is especially suited for biodefense agents as clinical evaluation of efficacy is usually not possible or ethical. The development of animal models that simulate human diseases by bioterror agents is of critical importance in this non-traditional route of drug repurposing for biodefense use. New avenues to be considered include the use of FDA-approved drugs singularly, in combination, or in tandem. In the case of simultaneous dosing, a lower dose of each drug with different mechanisms of action might produce synergistic beneficial effects and limit individual drug toxicity. Drugs used in tandem will likely be cooperative with the first drug muting out the early host inflammatory response and the second drug acting on secondary signals. Systematic identification of novel synergistic drug combinations will be beneficial to treat a multi-system and complex disease such as TSS.

## 10. Summary

Significant advances have been made in cell activation signals and pathways induced by staphylococcal superantigens. The superantigenic properties of SEB make it an “ideal” toxin to study the cellular interactions, biological effects and therapeutic interventions. Newer mouse models of toxic shock using human HLA class II transgenic mice or SEB un-potentiated mice can better define the systemic effects of SEB and aid in the therapeutic discovery to prevent TSS. Targeting proinflammatory mediators and T-cell cytokines appears to be most beneficial yet not all anti-inflammatory drugs are effective in preventing shock. The use of FDA-approved drugs, rational combinations of FDA-approved drugs, and changing treatment modality are avenues to fast-track and repurpose old drugs for biodefense use. Immunosuppressants, combinations of an immunosuppressant with an anti-oxidant and other carefully tailored combinations hold promise as treatment options for TSS.
